# Chronic insomnia is associated with inflammatory, carotid atherosclerotic, MRI-based brain atrophy-related, and cognitive alterations: the potential role of IL-6

**DOI:** 10.3389/fmed.2026.1868267

**Published:** 2026-08-13

**Authors:** Han Li, Rihong Wei, Ting Jiang, Mengdan Chu, Xiaoyin Jin, Zengfeng Su

**Affiliations:** Department of General Practice, The Fourth Affiliated Hospital of Anhui Medical University (Chaohu Hospital of Anhui Medical University), Chaohu, China

**Keywords:** atherosclerosis, brain atrophy, chronic insomnia disorder, cognitive impairment, interleukin-6, MMSE

## Abstract

**Objective:**

This study aimed to investigate the co-occurrence patterns of inflammatory, carotid atherosclerotic, MRI-based brain atrophy-related, and cognitive alterations in patients with Chronic Insomnia Disorder (CID), with a focus on exploring the statistical association of IL-6 with insomnia severity and cognitive function.

**Methods:**

A total of 88 patients with chronic insomnia and 60 healthy controls undergoing physical examinations were recruited from Chaohu Hospital of Anhui Medical University between May 2024 and April 2025. Insomnia severity was assessed using the Athens Insomnia Scale (AIS). Serum levels of white blood cell count (WBC), high-sensitivity C-reactive protein (hs-CRP), interleukin-6 (IL-6), and lipid profiles were measured using automated hematology and biochemistry analyzers. Carotid intima-media thickness (CIMT) and plaque incidence were evaluated via color Doppler ultrasound. Brain morphological changes were examined using magnetic resonance imaging (MRI), and global cognitive function was assessed with the Mini-Mental State Examination (MMSE). Differences in indicators between the two groups were compared. Pearson correlation analysis was used to examine the relationships among hs-CRP, IL-6, lipid levels, cognitive decline, and insomnia severity. Subsequently, Model 4 of the SPSS macro PROCESS (v4.1) was used as an exploratory cross-sectional path model. Because the exposure, mediator, and outcome were measured at the same time point, the model was interpreted as an association-based indirect-effect analysis rather than evidence of causal mediation.

**Results:**

(1) Compared to the control group, the chronic insomnia group had significantly higher levels of hs-CRP, IL-6, homocysteine, and triglycerides, and significantly lower levels of high-density lipoprotein (all *P* < 0.05). (2) Both CIMT and carotid plaque incidence were higher in the chronic insomnia group than in the control group (L-CIMT: 0.89 ± 0.22 vs. 0.74 ± 0.17; R-CIMT: 0.90 ± 0.24 vs. 0.76 ± 0.15; plaque incidence: 29.0% vs. 15.0%). (3) The frontal lobe b/i ratio was slightly higher in the chronic insomnia group, but the difference was not statistically significant. Significant differences were found between the two groups in the cerebellar a/h ratio, temporal lobe e/k ratio, average hippocampal width, average sulcal width, and MMSE scores (all *P* < 0.05). (4) Pearson analysis showed that in the chronic insomnia group, IL-6 and triglyceride levels were positively correlated with AIS scores (*P* < 0.05) and negatively correlated with MMSE scores (*P* < 0.05). (5) The exploratory path model indicated a significant positive association between triglyceride (TG) levels and AIS scores (R = 0.36, *P* < 0.05). TG levels showed a negative but non-significant predictive effect on MMSE scores (B = −0.96, *P* > 0.05). Testing IL-6 in the AIS-MMSE path framework revealed a significant positive association with AIS scores (R = 0.30, *P* < 0.05), and IL-6 was significantly associated with MMSE scores in the regression model (B = −0.16, *P* < 0.05). Further indirect-effect analysis showed a direct effect of −0.1534 (accounting for 74.63% of the total effect) and an indirect effect of −0.0528 (accounting for 25.37% of the total effect), with a total effect of −0.2052. These results should be interpreted cautiously because of the cross-sectional design. (6) Patients with CID simultaneously exhibited elevated inflammatory levels, carotid atherosclerosis, MRI-based brain atrophy-related changes, and lower cognitive screening scores. This pattern suggests that CID is associated with multi-system clinical and biological alterations. IL-6 was statistically involved in the AIS-MMSE association in an exploratory cross-sectional path model. Because aging is a complex process involving multiple biological pathways beyond inflammaging, vascular aging, brain atrophy, and cognition, these findings should be interpreted as evidence of associated biomarker patterns rather than proof that CID globally accelerates biological aging or directly causes neurodegeneration.

## 1 Introduction

Chronic Insomnia Disorder (CID) is a persistent sleep disorder characterized by difficulties in sleep initiation and/or maintenance, dissatisfaction with sleep duration or quality, and daytime dysfunction lasting for more than 3 months. It is one of the most common public health problems (1). Beyond psycho-neurological symptoms, CID has been associated with cardiovascular disease, metabolic abnormalities, immune dysregulation, and cognitive impairment (2, 3), suggesting that its clinical consequences may extend across multiple physiological systems.

In this study, CID was examined within a multi-system clinical biomarker framework. Aging-related clinical vulnerability can be reflected by changes across several biological domains, including chronic low-grade inflammation (inflammaging), vascular structural remodeling, cerebral structural change, and decline in cognitive reserve (4–7). However, these markers should not be equated with direct measurement of biological age. Therefore, CID may not only represent a sleep disorder, but may also be associated with a multi-system pattern of inflammatory, vascular, brain structural, and cognitive alterations.

First, inflammation may be an important biological correlate linking insomnia to aging-related clinical vulnerability. Increasing evidence indicates that sleep disturbance is associated with elevated inflammatory biomarkers, especially IL-6 and high-sensitivity C-reactive protein (hs-CRP) (8–11). IL-6 is regarded as a pivotal mediator of inflammaging because it participates in immune-metabolic dysregulation and can promote vascular endothelial dysfunction, neuroinflammation, and tissue degeneration (4). Nevertheless, in the present cross-sectional study, IL-6 should be interpreted as an associated inflammatory marker rather than proof of a mechanistic pathway.

Second, inflammatory and metabolic changes associated with CID may contribute to carotid atherosclerosis. Composite hematological inflammatory indices, including the systemic immune-inflammation index (SII), platelet-to-lymphocyte ratio (PLR), neutrophil-to-lymphocyte ratio (NLR), and systemic inflammation response index (SIRI), have been linked to sleep disorders in prior studies (12–15). Homocysteine (Hcy), which is closely related to inflammation and oxidative stress, may also interfere with neurotransmitter regulation and vascular homeostasis (16). In addition, population-based evidence has connected sleep disturbance, inflammation, and cognitive function, as well as sleep duration and inflammatory biomarkers (17, 18). These changes may be reflected clinically by increased carotid intima-media thickness (CIMT) and plaque formation, both of which are recognized markers of vascular aging and atherosclerosis (19, 20).

Third, CID may be associated with MRI-based brain atrophy-related measurements. Previous MRI studies have shown that patients with insomnia can exhibit reduced hippocampal volume and gray matter changes in frontal and temporal regions (21–23). Such structural abnormalities may arise from chronic sleep fragmentation, persistent inflammation, impaired glymphatic clearance, and vascular insufficiency. In the present study, the imaging indices were used as clinically practical structural markers and should not be interpreted as definitive evidence of neurodegeneration or biological brain-age acceleration.

Finally, vascular changes, brain structural alterations, and inflammatory activation may be associated with cognitive performance. Sleep disturbance, inflammatory cytokines, metabolic disturbance, and vascular remodeling have all been associated with cognitive impairment or dementia risk (17, 24). Therefore, the objective of this study was to investigate the co-occurrence patterns of inflammatory, carotid atherosclerotic, MRI-based brain atrophy-related, and cognitive alterations in patients with CID, with a focus on exploring the statistical association of IL-6-related inflammation with insomnia severity and cognitive function.

## 2 Research subjects and methods

### 2.1 Research subjects

This cross-sectional case-control study enrolled 88 patients with CID from the Sleep Medicine and General Medicine departments of The Fourth Affiliated Hospital of Anhui Medical University (Chaohu Hospital of Anhui Medical University) between May 2024 and April 2025. During the same period, 60 healthy volunteers were recruited from the hospital health check-up center as the control (CO) group. Controls were frequency-matched to the CID group by age, gender, ethnicity, and education level. Participants were allocated to the CID or CO group according to the following predefined criteria. Because the study compared a predefined CID group with a healthy control group, the results primarily describe differences between selected groups and should not be interpreted as a dose-response relationship across the full insomnia-symptom continuum in the general population.

Inclusion criteria for the CID group: (1) Diagnosis of non-organic insomnia according to the International Classification of Diseases, 10th edition (ICD-10); (2) Insomnia duration > 3 months; (3) Athens Insomnia Scale (AIS) score > 6; (4) Hamilton Anxiety Scale score ≤ 4 and Hamilton Depression Scale score ≤ 7; (5) Age 50–70 years; (6) Educational duration ≥5 years; (7) No clinically diagnosed cognitive impairment or hearing impairment that would prevent assessment; (8) Voluntary participation with signed informed consent.

Inclusion criteria for the CO group: (1) No history of insomnia or long-term sleep disturbance; (2) No anxiety, depression, or cognitive disorder; (3) Normal language communication ability and ability to complete the questionnaires and examinations; (4) Age, gender, ethnicity, and education level comparable to those of the CID group; (5) Voluntary participation with signed informed consent.

Exclusion criteria for all participants: (1) Definite neuropsychiatric disorders, including Parkinson's disease, schizophrenia, bipolar disorder, depression, anxiety disorder, or obsessive-compulsive disorder; (2) Severe endocrine disease, such as pituitary tumor, adrenal tumor, or hyperthyroidism; (3) Diagnosed cerebrovascular disease; (4) Infection or inflammatory disease within the preceding 2 weeks; (5) Secondary insomnia caused by other physical or mental conditions; (6) Use of sleep-related medication within the preceding 2 weeks; (7) Autoimmune disease or acute/chronic inflammatory disease; (8) Obstructive sleep apnea; (9) Refusal or inability to provide written informed consent.

### 2.2 Research methods

#### 2.2.1 General information

Demographic data were collected, including gender, age, and years of education. All participants completed the Athens Insomnia Scale (AIS) before enrollment. AIS was used consistently for insomnia screening, descriptive statistics, correlation analysis, and exploratory path analysis in the present study. Because AIS scores were highly separated between the CID and control groups by design, analyses using continuous AIS scores should be interpreted cautiously.

The Athens Insomnia Scale (AIS) (25) was used to assess the severity of insomnia. The scale primarily measures difficulty falling asleep, maintaining sleep, early awakening, and the subjective experience of sleep quality. The AIS consists of 8 items, with the first 5 items assessing sleep difficulties and the last 3 items assessing daytime functional impairments. Each item is scored from 0 to 3 points, with a total score ranging from 0 to 24. A score < 4 indicates no insomnia, a score between 4–6 suggests possible insomnia, and a score >6 indicates insomnia.

The Mini-Mental State Examination (MMSE) (26) was used to assess global cognitive function. The test consists of 20 questions with 30 items in total. Each correct answer is scored 1 point, and incorrect answers receive 0 points. The total score ranges from 0 to 30. The cutoff for normal and abnormal scores varies according to education level: for those with ≤ 6 years of education, a score ≤ 20 is considered abnormal; for those with >6 years of education, a score ≤ 24 is considered abnormal. Lower scores indicate poorer cognitive screening performance. In this study, MMSE was used as a brief global cognitive screening tool rather than a diagnostic neuropsychological battery. Participants were not clinically diagnosed with cognitive impairment at enrollment and were able to complete the assessment and provide written informed consent; nevertheless, the MMSE results should be interpreted cautiously.

#### 2.2.2 Blood routine and biochemical indicators

A full-automatic blood cell analyzer was used to measure white blood cell (WBC) count, platelet count, lymphocyte count, neutrophil count, and monocyte count. A full-automatic biochemical analyzer was used to measure hs-CRP, IL-6, total cholesterol (TC), triglycerides (TG), low-density lipoprotein cholesterol (LDL-Ch), high-density lipoprotein cholesterol (HDL-Ch), and homocysteine (Hcy). The hematological inflammatory indices were calculated as follows: PLR = platelet count/lymphocyte count; NLR = neutrophil count/lymphocyte count; SII = platelet count × neutrophil count/lymphocyte count; and SIRI = neutrophil count × monocyte count/lymphocyte count.

#### 2.2.3 Carotid ultrasound examination methods and standards

Participants were asked to lie on their backs with their heads supported. A routine examination of the left and right common, internal, and external carotid arteries was performed. Maximum carotid intima-media thickness (CIMT) was measured, and the presence of atherosclerotic plaques and their degree of stenosis were assessed. CIMT ≥0.10 cm at the carotid bifurcation was considered thickening of the intima-media, which is the standard for diagnosing arteriosclerosis. CIMT > 1.5 mm or localized intima thickness greater than 50% of the surrounding intima thickness was defined as a plaque.

#### 2.2.4 Brain atrophy measurement

Linear measurements and visual inspection were used to assess cerebral atrophy-related structural changes on cranial MRI scans. The frontal, temporal (hippocampal), parietal, occipital, and cerebellar regions were visually inspected for atrophy. Linear measurements were performed by the same radiologist using fixed brain diameter indices. These measures were selected because they are clinically practical, but they are less precise than automated volumetric or cortical-thickness methods. Therefore, they were interpreted as MRI-based atrophy-related indices rather than direct measures of neurodegeneration or biological brain age. The following indices were calculated:

(1) Fourth ventricle maximum horizontal diameter (a), posterior fossa maximum horizontal diameter (h), and fourth ventricle index (a/h), indicating cerebellar atrophy;
(2) Maximum distance between the two frontal horns of the lateral ventricles (b), horizontal brain diameter (i), and frontal horn index (b/i), indicating frontal lobe atrophy;
(3) Minimum distance between the lateral walls of the lateral ventricular body (e), horizontal brain diameter (k), and lateral ventricular body index (e/k), indicating temporal lobe atrophy;
(4) Hippocampal width measured at the widest point;
(5) Mean width of the four widest cerebral sulci on the second-to-last imaging slice, indicating parietal lobe atrophy.

### 2.3 Statistical methods

SPSS 27 statistical software was used for data analysis. Descriptive statistics: Normally distributed continuous data were presented as (x ± s), non-normally distributed continuous data were expressed as M [P25, P75], and categorical data were presented as [*n* (%)].

#### 2.3.1 Group comparisons

For continuous data with equal variances and normal distribution, an independent *t*-test was used. For data with unequal variances or non-normal distribution, the rank-sum test was used. Categorical data were analyzed using the chi-square test. Pearson's correlation method was used for correlation analysis. PROCESS (v4.1) Model 4 was used only as an exploratory cross-sectional path model to estimate statistical indirect effects. No causal inference or temporal mediation was assumed. Because multiple biomarkers, imaging indices, and correlation analyses were examined, the findings should be considered exploratory; no formal multiple-testing correction was applied. A *P*-value < 0.05 was considered statistically significant.

## 3 Results

### 3.1 Comparison of general data and AIS scores between the two groups

The results showed that there were no significant differences between the CID group and the control group in terms of age, gender composition, and education level (*P* > 0.05). However, the difference in AIS scores was statistically significant (*P* < 0.05), as shown in Table 1. This large difference reflects the predefined case-control grouping strategy and should not be interpreted as representing the full distribution of insomnia severity in the general population.

**Table 1:** Comparison of general clinical data between the two groups [*n* (%), (*x* ± *s*)].

Group	*N*	Age (years)	Gender (male/female)^a^	Years of education	AIS score
Control group	60	57.6 ± 6.7	26/34	8.5 ± 3.2	4.0 ± 1.3
Chronic insomnia group	88	59.5 ± 9.7	38/50	7.4 ± 4.3	15.9 ± 3.4
z/*x*^2^ values		2.7	7.7	3.0	22.5
*P* values		0.43	0.37	0.34	< 0.001

^a^The χ^2^ value for the comparison of gender between the two groups was tested using the χ^2^ test, and other values were tested using the Mann-Whitney U test.

### 3.2 Comparison of inflammatory-related indicators and lipid levels between the two groups

As shown in Table 2, the CID group had significantly higher hs-CRP, IL-6, TG, and Hcy levels than the CO group, whereas HDL-Ch levels were significantly lower (all *P* < 0.05). These results indicate that CID was accompanied by both cytokine/acute-phase inflammatory activation and Hcy-related inflammatory-metabolic disturbance. In contrast, WBC, PLR, NLR, SII, and SIRI were slightly higher or comparable in the CID group but did not differ significantly between groups (all *P* > 0.05). Therefore, among the inflammatory markers examined in this study, IL-6, hs-CRP, and Hcy showed the clearest group differences, whereas the cell-count-derived inflammatory ratios did not.

**Table 2:** Comparison of cytokine, acute-phase, hematological inflammatory, and lipid-related indicators between the two groups [*n* (%), *x* ± *s*, or M (P25, P75)].

Indicator	Control group	Chronic insomnia group	*t*/z values	*P* values
WBC	5.37 ± 1.15	5.68 ± 1.66	2.392	0.457
hs-CRP	1.27 ± 0.79	2.34 ± 2.00	12.808	0.008
IL-6	1.52 (1.50, 1.79)	3.32 (2.54, 6.35)	−4.636	< 0.001
TC	4.45 ± 0.47	4.39 ± 0.90	11.039	0.733
TG	1.11 ± 0.30	1.69 ± 0.84	11.512	0.001
LDL-Ch	2.60 ± 0.47	2.37 ± 0.81	7.646	0.202
HDL-Ch	1.27 ± 0.25	1.10 ± 0.32	0.353	0.044
Hcy	9.83 ± 1.77	12.27 ± 4.80	4.735	0.013
PLR	128.09 ± 34.18	127.78 ± 36.93	0.152	0.947
NLR	1.93 ± 0.47	2.14 ± 0.81	4.307	0.263
SII	408.97 ± 108.34	440.12 ± 209.65	16.016	0.495
SIRI	0.65 (0.45, 0.73)	0.74 (0.42, 1.03)	0.607	0.551

WBC, white blood cell count; hs-CRP, high-sensitivity C-reactive protein; IL-6, interleukin-6; TC, total cholesterol; TG, triglycerides; LDL-Ch, low-density lipoprotein cholesterol; HDL-Ch, high-density lipoprotein cholesterol; Hcy, homocysteine; PLR, platelet-to-lymphocyte ratio; NLR, neutrophil-to-lymphocyte ratio; SII, platelet count × neutrophil count/lymphocyte count; SIRI, neutrophil count × monocyte count/lymphocyte count.

### 3.3 Comparison of CIMT and carotid artery plaque incidence between the two groups

The results indicated that both CIMT and carotid artery plaque incidence were higher in the chronic insomnia group than in the control group (L-CIMT: 0.89 ± 0.22 vs. 0.74 ± 0.17; R-CIMT: 0.90 ± 0.24 vs. 0.76 ± 0.15; plaque incidence: 29.0% vs. 15.0%). See Table 3.

**Table 3:** Comparison of bilateral CIMT and carotid artery plaque incidence between the two groups.

Variable	Control group	CID group	*t*/χ^2^ values	*P* values
L-CIMT (mm)	0.74 ± 0.17	0.89 ± 0.22	−2.295	0.027
R-CIMT (mm)	0.76 ± 0.15	0.90 ± 0.24	−2.325	0.030
Plaque incidence [*n* (%)]	3 (0.15)	10 (0.29)	4.546	0.033

### 3.4 Comparison of brain diameter ratios and MMSE scores between the two groups

The results showed that the frontal lobe b/i ratio in the CID group was slightly higher than that in the control group, but the difference was not statistically significant. However, there were statistically significant differences between the two groups in cerebellum a/h ratio, temporal lobe e/k ratio, hippocampal average width, average brain sulcus width, and MMSE scores. These MRI-based linear measurements were interpreted as atrophy-related structural indices rather than definitive measures of neurodegeneration. See Table 4.

**Table 4:** Comparison of brain diameter ratios and MMSE scores between the two groups.

Clinical indicator	Control group	Chronic insomnia group	*t*/*x*^2^ values	*P* values
Cerebellum a/h ratio	0.13 ± 0.01	0.24 ± 0.07	19.087	< 0.001
Frontal lobe b/i ratio	0.28 ± 0.08	0.30 ± 0.19	8.666	0.109
Temporal lobe e/k ratio	0.13 ± 0.01	0.22 ± 0.04	11.918	< 0.001
Hippocampal average width	15.85 ± 1.00	14.93 ± 0.64	2.277	< 0.001
Average brain sulcus width	2.24 ± 0.36	3.00 ± 0.34	0.057	< 0.001
MMSE score	25.45 ± 2.95	23.32 ± 3.63	2.636	0.030

Cerebellar a/h ratio = Maximum transverse diameter of the fourth ventricle (a)/Maximum transverse diameter of posterior fossa brain tissue (h). Frontal lobe b/i ratio = Widest distance between bilateral frontal horns of lateral ventricles (b)/Transverse diameter of brain tissue at level b (i). Temporal lobe e/k ratio = Minimal distance between bilateral lateral walls of lateral ventricular bodies (e)/Transverse diameter of brain tissue at level e (k).

### 3.5 Correlation analysis of MMSE scores and Hcy levels with brain diameter measurements

Pearson analysis showed that MMSE were negatively correlated with brain diameter measurements (cerebellum a/h ratio, frontal lobe b/i ratio, temporal lobe e/k ratio, and average brain sulcus width). Hcy levels were positively correlated with cerebellum a/h ratio, temporal lobe e/k ratio, and average brain sulcus width. No correlation was observed between MMSE, Hcy levels, and hippocampal average width. See Table 5.

**Table 5:** Correlation analysis of MMSE scores and Hcy levels with brain diameter measurements.

Variable	MMSE	Hcy	Cerebellum a/h ratio	Frontal lobe b/i ratio	Temporal lobe e/k ratio	Hippocampal average width
Hcy	−0.140					
Cerebellum a/h ratio	−0.623^**^	0.404^**^				
Frontal lobe b/i ratio	−0.328^*^	0.046	0.358^*^			
Temporal lobe e/k ratio	−0.501^**^	0.393^*^	0.852^**^	0.335^*^		
Hippocampal average width	−0.033	−0.033	−0.243	−0.140	−0.382^*^	
Average brain sulcus width	−0.353^*^	0.305^*^	0.651^**^	0.394^**^	0.773^**^	−0.351^*^

^*^P < 0.05, ^**^P < 0.01.

### 3.6 Correlation analysis between blood-related indicators and MMSE scores, AIS scores

Pearson analysis showed that in the CID group, IL-6 and triglycerides were positively correlated with AIS scores (*P* < 0.05); IL-6 and triglycerides were negatively correlated with MMSE scores (*P* < 0.05); hs-CRP levels were significantly negatively correlated with MMSE scores (*P* < 0.05), while HDL-Ch levels were positively correlated with MMSE scores (*P* < 0.05). See Table 6.

**Table 6:** Correlation analysis between blood indicators and MMSE scores, AIS scores.

Variable	AIS	MMSE	hs-CRP	IL-6	Triglycerides
MMSE	−0.375^**^				
hs-CRP	0.259	−0.372^**^			
IL-6	0.299^*^	−0.401^**^	0.095		
Triglycerides	0.363^**^	−0.308^*^	0.292^*^	0.300^*^	
HDL-Ch	−0.262	0.367^**^	−0.206	−0.164	−0.496^**^

^*^P < 0.05, ^**^P < 0.01.

### 3.7 Exploratory cross-sectional path analysis

**Figure 1:**
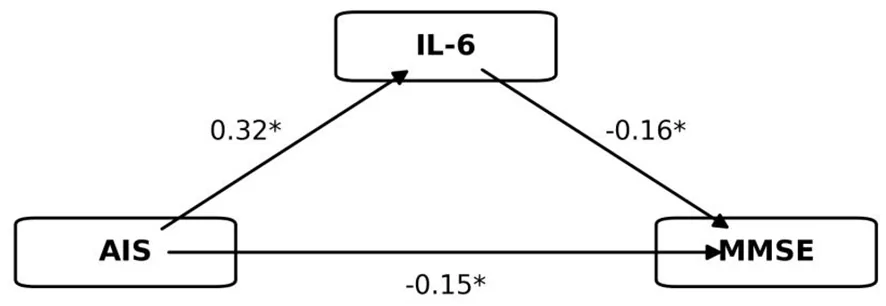
Exploratory cross-sectional path model. AIS scores were used as the independent variable, IL-6 as the intermediate variable in the path model, and MMSE scores as the dependent variable. Path coefficients are standardized regression coefficients. This figure represents an exploratory statistical path model based on cross-sectional data and should not be interpreted as proof of causal mediation. *P < 0.05.

Before data analysis, all continuous variables were standardized; therefore, the path coefficients shown in Tables 7, 8 and Figure 1 are standardized regression coefficients. Then, PROCESS (v4.1) Model 4 was used as an exploratory cross-sectional path model, with path coefficients shown in Tables 7–9 and Figure 1. TG was also examined because it was correlated with both AIS and MMSE scores. Although TG was positively associated with AIS scores, its predictive effect on MMSE scores was not statistically significant; therefore, a bootstrap indirect-effect table is presented only for IL-6. The test results for IL-6 showed that IL-6 was significantly positively associated with AIS scores; in the MMSE regression model, IL-6 was also significantly associated with MMSE scores. Further analysis of the indirect effect revealed that the direct effect was −0.1534, accounting for 74.63% of the total effect; the indirect effect was −0.0528, accounting for 25.37%. The total effect was −0.2052. Because all variables were measured cross-sectionally, these results describe statistical associations and should not be interpreted as evidence that insomnia causes IL-6 elevation or that IL-6 causally mediates cognitive impairment.

**Table 7:** Exploratory regression analysis of triglycerides in the AIS-MMSE framework.

Result variable	Predictor	Model fit indices			Regression coefficients	
		R	R^2^	F	B coefficient	*t* value
TG	AIS scores	0.36	0.13	7.31	0.04	2.70^*^
MMSE scores	AIS scores	0.41	0.17	4.77	−0.16	−2.05^*^
	TG				−0.96	−1.41

Model fit indices are presented as R, R^2^, and F. B indicates the regression coefficient; ^*^P < 0.05.

**Table 8:** Exploratory IL-6 path model in the relationship between AIS scores and cognitive function.

Result variable	Predictor	Model fit indices			Regression coefficients	
		R	R^2^	F	B coefficient	*t* value
IL-6	AIS scores	0.30	0.09	5.10	0.32	2.26^*^
MMSE scores	AIS scores	0.48	0.23	7.71	−0.15	−2.18^*^
	IL-6				−0.16	−2.46^*^

Model fit indices are presented as R, R^2^, and F. B indicates the standardized regression coefficient because continuous variables were standardized; ^*^P < 0.05.

**Table 9:** Exploratory indirect-effect analysis of IL-6 in the relationship between AIS scores and cognitive function.

Item	Effect value	SE values	95%CI		Effect proportion (%)
			Lower limit	Upper limit	
Direct effect	−0.1534	0.0703	−0.295	−0.012	74.63%
Indirect effect	−0.0528	0.0832	−0.302	−0.039	25.37%
Total effect	−0.2052	0.0703	−0.346	−0.064	100%

## 4 Discussion

This study investigated whether CID is accompanied by a coordinated pattern of inflammatory, carotid atherosclerotic, MRI-based brain atrophy-related, and cognitive alterations, and whether IL-6 is statistically involved in the association between insomnia severity and cognitive function. The principal findings were that CID patients showed: (1) elevated inflammatory biomarkers, particularly IL-6, hs-CRP, and Hcy; (2) increased CIMT and carotid plaque incidence, indicating carotid atherosclerotic changes; (3) MRI-based atrophy-related structural differences in multiple regions; and (4) lower MMSE scores. These findings suggest that CID may be associated with multi-system clinical and biological alterations rather than isolated sleep symptoms. However, given the cross-sectional and selected case-control design, the results should be interpreted as associative and exploratory.

### 4.1 Multi-system biomarker pattern in CID

The present study uses the term multi-system biomarker pattern as a descriptive framework rather than as direct evidence of global biological age acceleration. In the CID group, inflammatory activation, vascular morphological changes, brain structural changes, and lower cognitive screening performance co-occurred. Although a cross-sectional study cannot establish temporal causality, the simultaneous presence of these markers suggests that CID may be associated with alterations across immune, vascular, and central nervous systems. This interpretation is consistent with recent epigenetic-clock studies reporting that insomnia symptoms, short sleep, or poor sleep quality are associated with accelerated DNA-methylation-based aging measures such as GrimAge and DunedinPACE (27, 28). However, because epigenetic clocks were not measured in our cohort, our findings should be considered complementary clinical biomarker evidence rather than direct molecular evidence of accelerated aging.

### 4.2 IL-6-related inflammation as a central hub

CID patients had significantly higher IL-6 and hs-CRP levels than controls, indicating activation of a chronic low-grade inflammatory state. This is consistent with the concept of inflammaging, in which persistent inflammatory signaling contributes to age-related immune-metabolic dysregulation and tissue dysfunction (4, 29). Hcy was also significantly elevated in the CID group, suggesting an additional inflammatory-metabolic component that may influence vascular and neural injury. By contrast, PLR, NLR, SII, and SIRI did not differ significantly between groups. This pattern indicates that, in this cohort, soluble inflammatory mediators and Hcy were more sensitive indicators of CID-related inflammatory burden than cell-count-derived ratios.

### 4.3 Carotid atherosclerosis-related vascular findings

At the vascular level, CID patients showed increased left and right CIMT and a higher plaque incidence. CIMT is a widely used structural marker of early atherosclerosis and vascular risk. The concurrent elevation of IL-6 and hs-CRP provides a plausible biological association, because inflammatory cytokines may promote endothelial dysfunction, lipid deposition, arterial wall remodeling, and plaque formation (5, 19, 20). These results suggest that CID may be associated with early carotid atherosclerotic changes, potentially through inflammatory and metabolic pathways. Nevertheless, CIMT and plaque incidence are clinical vascular-risk markers and should not be interpreted as direct measurements of biological aging.

### 4.4 MRI-based brain atrophy-related indices and cognitive screening

At the brain structural level, the CID group showed higher cerebellar a/h ratio, higher temporal lobe e/k ratio, wider brain sulci, and reduced hippocampal width, together with lower MMSE scores. These findings indicate that MRI-based brain atrophy-related indices and cognitive screening performance may differ between selected CID patients and healthy controls. Peripheral inflammatory activation may contribute to this process by increasing blood-brain barrier permeability, activating microglia, promoting neuroinflammation, impairing synaptic plasticity, and reducing neuronal integrity (6, 30–32). In addition, insomnia itself may impair glymphatic clearance and memory consolidation, which could further contribute to cognitive vulnerability (33, 34). However, the linear MRI indices used here are relatively crude structural measures, and MMSE is a brief screening instrument. Therefore, these findings cannot by themselves establish neurodegeneration or clinically diagnosed cognitive disorder.

### 4.5 Exploratory role of IL-6 and clinical implications

The exploratory path analysis showed that IL-6 was statistically involved in the relationship between insomnia severity and cognitive function, accounting for 25.37% of the total effect in the cross-sectional model. This finding supports the possibility that inflammation is one relevant correlate of cognitive performance in CID. However, because all variables were measured at the same time point and major confounders could not be fully adjusted, the result should not be interpreted as causal mediation. TG was correlated with both AIS and MMSE scores, suggesting that metabolic disturbance may accompany insomnia-related cognitive and vascular changes. However, TG did not show a statistically significant predictive effect in the MMSE regression model; therefore, it should be interpreted as an associated metabolic indicator rather than a significant intermediate variable in the current cohort. Clinically, these results suggest that evaluation of CID in middle-aged and older adults may benefit from attention to inflammatory, metabolic, vascular, brain structural, and cognitive indicators, while stronger causal claims require longitudinal and more fully adjusted studies.

## 5 Limitations

This study has several limitations that should be considered when interpreting the findings:

First, the cross-sectional design is a primary constraint. Although we used PROCESS Model 4 to explore the potential statistical indirect effect involving IL-6, the exposure, intermediate variable, and outcome were measured at the same time point. Therefore, the model cannot determine temporal order or causal mediation among chronic insomnia, inflammatory activation, vascular/brain structural changes, and cognitive decline. For example, pre-existing brain atrophy or vascular pathology may themselves cause or exacerbate sleep disturbances.

Second, the study used a selected case-control design. The CID group and control group showed highly separated AIS scores, which is expected given the inclusion criteria. Thus, the group comparisons describe differences between selected CID patients and healthy controls, but they do not establish a dose-response relationship across the full insomnia-symptom continuum in the general population. The sample size was also relatively limited and single-centered, which may restrict generalizability and hinder more detailed subgroup analyses.

Third, there are limitations related to confounding and multiple testing. Although age, gender, ethnicity, and education were frequency-matched, several important cardiometabolic, psychiatric, medication-related, socioeconomic, and behavioral factors, such as BMI, smoking, hypertension, diabetes, cardiovascular history, physical activity, and medication exposure, were not fully incorporated into the statistical models. In addition, many biomarkers, imaging indices, correlations, and exploratory path relationships were examined without formal multiple-testing correction. Therefore, the findings should be considered exploratory and hypothesis-generating.

Fourth, there are limitations in the measurement of brain structure and cognition. For the assessment of brain atrophy-related changes, we employed clinically practical linear MRI measurements and visual inspection; these methods are less sensitive than automated volumetric, cortical-thickness, or hippocampal segmentation approaches, and they cannot directly quantify biological brain age or neurodegeneration. MMSE was used as a brief global cognitive screening tool rather than a comprehensive neuropsychological battery. Although enrolled participants had no clinically diagnosed cognitive impairment that prevented participation and provided written informed consent, the relatively low MMSE scores indicate that future studies should include more detailed cognitive testing and clearer capacity-related documentation. Finally, the evaluation of aging has not yet incorporated molecular indicators such as epigenetic clocks, which can directly quantify biological age and have recently been linked to insomnia and poor sleep quality (27, 28).

## 6 Conclusion

Aligned with the revised manuscript title and study objective, this study demonstrates that patients with CID exhibit a co-occurring pattern of inflammatory activation, carotid atherosclerotic changes, MRI-based brain atrophy-related structural alterations, and lower cognitive screening performance. IL-6 was statistically involved in the association between insomnia severity and MMSE scores in an exploratory cross-sectional path model, suggesting that inflammation may be one relevant correlate linking CID with cognitive dysfunction. These findings should be interpreted as associative rather than causal and do not prove that CID directly accelerates biological aging or neurodegeneration. Longitudinal studies incorporating molecular aging indicators, detailed neuropsychological assessment, automated neuroimaging quantification, and more complete confounder adjustment are needed to clarify the clinical significance and causal direction of these associations.

## Data Availability

The original contributions presented in the study are included in the article/supplementary material; further inquiries can be directed to the corresponding author.
